# Prevalence and risk factors of *Toxoplasma gondii* infection in dairy cattle from the Western Region of Thailand[Fn FN1]

**DOI:** 10.1051/parasite/2024038

**Published:** 2024-07-09

**Authors:** Napasaporn Wannapong, Preeda Lertwatcharasarakul, Theera Rukkwamsuk

**Affiliations:** 1 Department of Large Animal and Wildlife Clinical Sciences, Faculty of Veterinary Medicine, Kasetsart University Kamphaeng Saen Nakhon Pathom 73140 Thailand; 2 Department of Pathology, Faculty of Veterinary Medicine, Kasetsart University Kamphaeng Saen Nakhon Pathom 73140 Thailand

**Keywords:** Dairy cattle, Risk factor, Serology, *Toxoplasma gondii*, Thailand

## Abstract

In total, 901 dairy cow sera and data were collected from 51 farms in Nakhon Pathom, Ratchaburi and Kanchanaburi provinces (Western Region of Thailand). Serum samples were processed via the multispecies ELISA method to detect IgG antibodies against *Toxoplasma gondii* infection. The results demonstrated that the calculated true prevalence was 1.48% (95% CI, 0.64–2.75%) for the individual-level and 29.41% (95% CI, 18.71–43%) for the farm-level. The univariate risk factor analysis showed that the number of total owned cats, the presence of stray cats, and the frequency of cleaning per day were significant factors (*p* < 0.2). These three factors were subjected to logistic regression analysis, and the results revealed that the frequency of cleaning farms per day was a potential risk factor for *T. gondii*-seropositive farms (OR = 2.745, 95% CI, 1.15–8.69, *p* = 0.02). The frequency of cleaning might increase the *T. gondii* oocyst distribution within the barn area, thus increasing the possibility of infection. Our findings show that *T. gondii* continues to circulate in the dairy cow population in the western part of Thailand. The presence of cats on farms was not found to be associated with *T. gondii* infection, but the high frequency of cleaning the floor was, and contributed to the potential risk of infection.

## Abstract (fr)

Au total, 901 sérums de vaches laitières et des données ont été collectés dans 51 fermes des provinces de Nakhon Pathom, Ratchaburi et Kanchanaburi (région occidentale de la Thaïlande). Les échantillons de sérum ont été traités via la méthode ELISA multi-espèces pour détecter les anticorps IgG contre l’infection à *Toxoplasma gondii*. Les résultats ont démontré que la prévalence réelle calculée était de 1,48 % (IC à 95 %, 0,64–2,75 %) au niveau individuel et de 29,41 % (IC à 95 %, 18,71–43 %) au niveau des exploitations. L’analyse factorielle a montré que le nombre total de chats possédés, la présence de chats errants et la fréquence quotidienne de nettoyage étaient des facteurs significatifs (*p* < 0,2). Ces trois facteurs ont été soumis à une analyse de régression logistique et les résultats ont révélé que la fréquence quotidienne de nettoyage des exploitations était un facteur de risque potentiel pour les exploitations séropositives à *T. gondii* (OR = 2,745, IC à 95 % = 1,15–8,69, *p* = 0,02). La fréquence du nettoyage pourrait favoriser la répartition des oocystes de *T. gondii* dans les étables, augmentant ainsi le risque d’infection. Nos résultats indiquent que *T. gondii* continue de circuler dans la population de vaches laitières de l’ouest de la Thaïlande. La présence de chats dans les fermes n’a pas été associée à l’infection à *T. gondii*, mais la fréquence élevée du nettoyage du sol l’était et contribuait au risque potentiel d’infection.

## Introduction

*Toxoplasma gondii* is an obligate intracellular protozoan that causes toxoplasmosis both in humans and mammals. The important clinical signs are abortion and neurological syndrome, which impact public health and the livestock industry. Hosts become infected by ingesting contaminated oocysts in food or water. Additionally, carnivorous hosts can be infected by consuming tissue cysts of the parasite in raw meat. The life cycle of *T. gondii* consists of both asexual and sexual cycles. The feline serves as the only definitive host, developing the sexual cycle by producing and shedding of un-sporulated oocysts into the environment via feces. Consequently, the sporulation of oocysts enables the protozoa to survive for long periods, even in extreme conditions due to the multilayered structure of the oocyst wall [23, 49]. The asexual cycle involves protozoan multiplication and differentiation between tachyzoites and bradyzoites. During the acute phase, tachyzoites invade enterocytes and subsequently enter the blood circulation. From there, the parasites disseminate throughout the body, infecting and multiplying within various host organs. The multiplication of parasites is decreased by the host immune response, which triggers tachyzoites to differentiate into bradyzoites and develop into tissue cysts or chronic stage. Conversely, when an infected host loses immune function, bradyzoites in tissue cysts can differentiate back to tachyzoites. This stage allows the parasite to disseminate throughout the body, causing re-emergence of the acute stage. Additionally, *T. gondii* infection can induce retinitis, myocarditis and placentitis, which can cause abortion in both humans and animals, including cattle. In pregnant hosts, fetuses may be infected through congenital transmission of tachyzoites [38].

The gold standard for *T. gondii* diagnosis is the dye test or Sabin–Feldman dye, which is an antibody detection method. However, it has disadvantages such as high cost and the use of live organisms, posing a human hazard. Other serological methods recommended for screening in survey studies include the indirect fluorescent antibody test (IFAT), modified agglutination test (MAT), indirect hemagglutination test (IHA), and latex agglutination test (LAT). Although these methods are convenient to perform, the results are interpreted visually, and individual variation may be a concern. One suitable technique for epidemiological studies is enzyme-linked immunosorbent assay (ELISA) due to its simplicity for mass screening surveys and accurate results read by a spectrophotometer. Viable parasites are detected by bioassay, with inoculation in a mouse or cat. Consequently, molecular techniques for genomic detection are necessary to confirm infection. These are time consuming processes requiring high biosecurity level in laboratories, making them unsuitable for survey studies [26, 43].

Dairy cattle are intermediate hosts of *T. gondii*. They become infected by ingesting contaminated oocysts in feed and water. The protozoa are then transferred to other hosts through the consumption of milk and undercooked meat from infected cattle [48]. Most studies have reported that cattle are resistant to *T. gondii*, and often infected pregnant cattle deliver normal calves and develop antibodies [9]. In addition, a study of pregnant cows (*n* = 4) showed that 50% of cows aborted after subcutaneous inoculation with tachyzoites [51]. In Thailand, the most recent survey was carried out 10 years ago and found a 9.42% seroprevalence in dairy cattle [20]. The western region is characterized by a high density of dairy cattle in Thailand, where the seroprevalence of *T. gondii* was 7% in 2008 [4]. Infection with *T. gondii* is not a specific clinical sign until cattle exhibit signs of fetal abortion and stillbirth. This is why it is difficult to diagnose toxoplasmosis in cattle. In addition, approximately one third of the global human population has been exposed to *T. gondii* [5, 38]. The foodborne nature of *T. gondii* via beef has not been confirmed, but viable protozoa have been detected from samples collected at slaughterhouses [32]. Although disease transmission from cattle to humans was not conclusively confirmed, *T. gondii* infection in cattle cannot be ruled out as a public health problem [10].

To prevent *T. gondii* infection in dairy cattle, several studies have investigated both risk and protective factors. Many findings suggest that prevention of contamination with oocysts is key. It is crucial to prohibit cats form accessing farm areas, including feed and water storage areas [19, 44]. Additionally, *T. gondii* infection is not a required test before animal transportation, and recently acquired animals should be screen tested and imported from reputable suppliers [3]. Differences in farm locations, management systems, and farmer activities are important considerations when implementing prevention strategies. This study aimed to determine the seroprevalence, spatial distribution, and risk factors for *T. gondii* infection in dairy cattle from the Western Region of Thailand. Understanding disease epidemiology is important for designing strategies for the control and prevention of infection.

## Materials and methods

### Ethics

This study was carried out with approval from the Committee for Animal Care and Use for Scientific Research, Kasetsart University, Thailand (ACKU64-VET-039). The farm owners gave permission for collection of samples and data.

### Study area, sample collection, and processing

**Figure 1:**
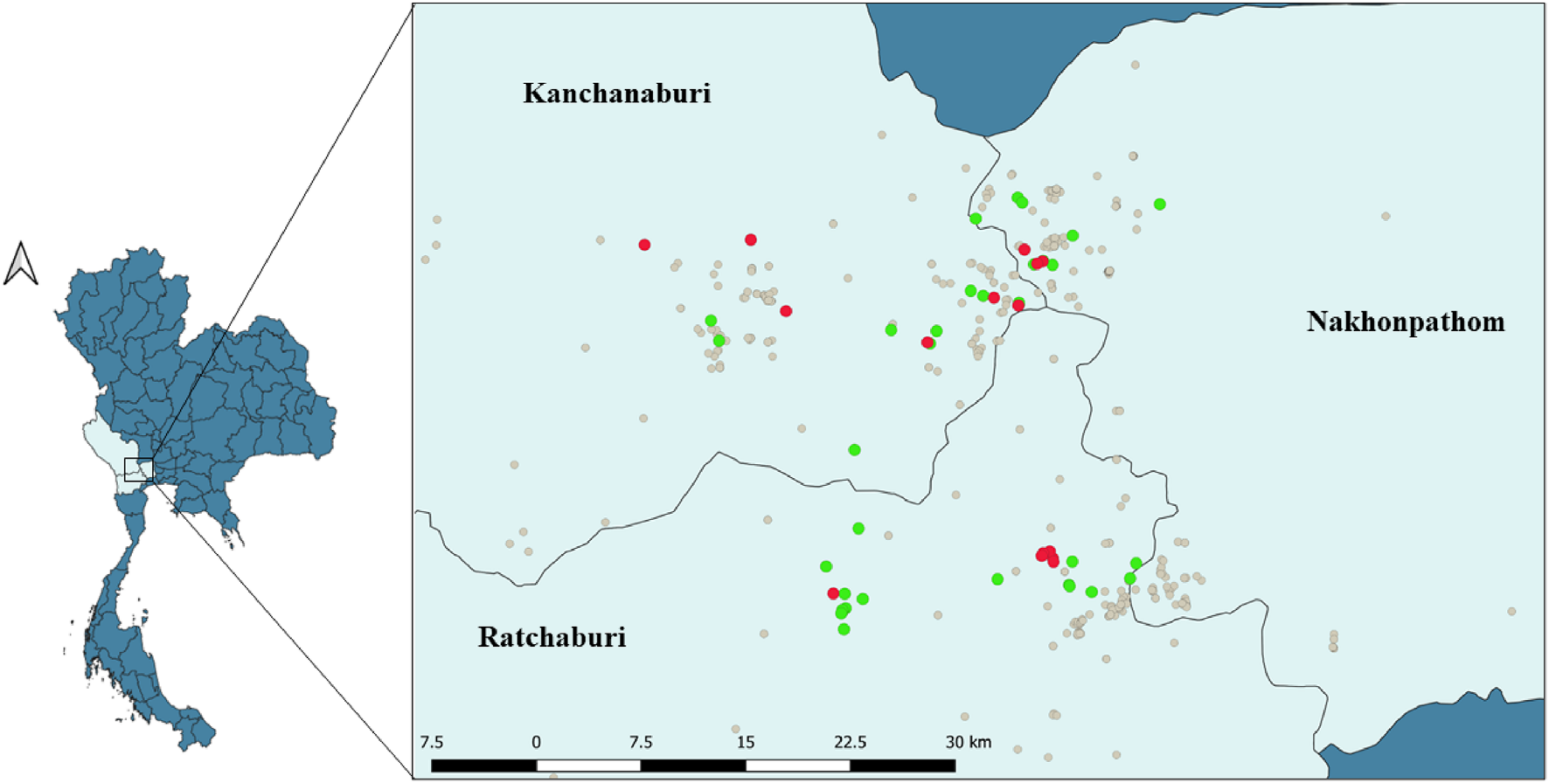
Study area in three provinces in the western part of Thailand. Geographical distribution of the dairy farms in this area, including *T. gondii* seropositive farms (red circles) and negative farms (green circles).

This study was carried out in three Western regions with the highest densities of dairy cows, namely Ratchaburi, Kanchanaburi and Nakhonpathom provinces (Fig. 1). The 51 farms were selected from members of the Kasetsart University Veterinary Teaching Hospital Kamphaeng Saen and Nong Pho, where mobile cinical services are provided in this area. The sample size of the seroprevalence study was calculated by the ProMESA program (version 2.3.0.2) via the estimated sample size–stratified random sample method. The calculation of the sample size required the value of the expected prevalence in each province, so we followed the 2010 study in Thailand; this value was 10% of the total *T. gondii* seroprevalence [20]. The population size in each province was calculated according to Department of Livestock data. In addition, the sample size (n) was calculated with an acceptable relative error of 0.2 and a 95% confidence interval following this formula.

$$n=\;\frac{\sum_{i=1}^{e}\left[\frac{{\;\left(n_{i}\right)}^{2}\times p_{i}\times \left(1-p_{i\;}\right)}{W_{i}}\;\right]}{N^{2\;}\times \frac{{\mathrm{AE}}^{2}}{Z^{2}}+\sum_{i=1}^{e}[n_{i\;}\times \left(1-p_{i\;}\right)]}$$

The calculated sample sizes were 388, 272, and 194 blood samples collected from Ratchaburi, Kanchanaburi and NakhonPathom, respectively. Then, the number of samples from each farm was estimated by the method of Thrusfield [47] for the detection of disease in a group of animals. The requested sample sizes for each farm were calculated using the following formula [47]:

$$n=[1-{\left(1-p_{1}\right)}^{\frac{1}{d}\;}]\;[N-d/2]\;+\;1$$

Approximately 4 mL of blood were collected from coccygeal vessels in randomly selected cattle. Blood samples were centrifuged, and the serum was separated then stored at −20 °C until analysis. The serodiagnosis of *T. gondii* infection was tested using an indirect ELISA multispecies diagnosis kit (ID Screen, ID.VET. *T. gondii*-specific IgG antibody, Innovative Diagnostics, Montpellier, France) [1]. Following the instructions of the manufacturer, the test presented 98% sensitivity and 99% specificity. The ELISA kit was applied to detect IgG antibodies, using the P30 (SAG1) antigen and the multispecies HRP conjugate antibody as the secondary antibody [27]. TMB substrate was added, and the reaction was then measured at 450 nm. The results were interpreted from S/P% calculation, using this formula [(OD _sample_ − OD_NC_)/(OD_PC_ − OD_NC_)] × 100. Samples with S/P% less than 40% were considered negative, those ranging from 40 to 50% doubtful, and those exceeding 50% positive. The doubtful cases were collected for new sample after 2 weeks and retested.

### Data collection

Data were obtained by interviewing and analyzed for risk factors associated with *T. gondii* seropositivity. This analysis included individual-level and herd-level analyses. The individual-level variables were as follows: dairy status (heifer/cow), parity, born in the farm (yes/no), abortion history during the past pregnancies (yes/no), and repeat breeding (yes/no). In addition, the herd-level variables included hospital member information (Kamphaeng Saen, KPS/Nong Pho, NP), location (Nakhon Pathom/Kanchanaburi/Ratchaburi), duration of farm, co-operative information (Nakhon Pathom, NKP/Sirichok, SR/Thamuang, TM/Nong Pho, NP), standard farm certification (yes/no), total number of cattle, farm type (tie stall/free-stall), presence of companion cats (yes/no), number of companion cats, presence of stray cats (yes/no), open stack of feed (yes/no), feeding with fresh grass (yes/no), pasture (yes/no), type of water (tap/ground/well), feeding area (ground/higher from the ground), frequency of cleaning per day, introduction of cattle from other farms (yes/no), and presence of soil or sand area in house (yes/no). These variables were used for interviewing farm owners.

### Statistical analysis

The estimated true prevalence (TP) was calculated considering the apparent prevalence (AP), sensitivity (Se), and specificity (Sp) of the test, as follow [40].

$$\mathrm{TP}\;=\;\frac{\mathrm{AP}+\mathrm{Sp}-1}{\mathrm{Se}+\mathrm{Sp}-1}$$

A farm was considered positive if at least one animal from the farm was infected. All the studied variables were analyzed by univariate analyses (binary logistic regression) at both individual and farm levels. Subsequently, if the *p* value of each analyzed variable was equal to or less than 0.2, the variable was considered to have sufficient significance for building a logistic regression model. The mixed effects of the significant variables were built for the final model by logistic regression analysis [44]. The associations between variables and seropositive farms were interpreted using odds ratio with 95% CI both in univariate and logistic regression analyses. All analyses were performed using RStudio software, R version 4.0.5, 2021-03-31 [33].

## Results

*Toxoplasma gondii* antibodies were detected in 22 out of the 901 serum samples; the apparent prevalence is 2.44% (95% CI, 1.55–3.399). The calculated true prevalence, derived from apparent prevalence, sensitivity and specificity of test, was 1.48% (95% CI, 0.64–2.75%). Seropositive rates across different parity ranged from 2.06 to 2.96%, showing closely similar percentages between each parity group. The univariate analysis between seropositive animal and factors presented no variables that cannot be included for logistic regression analysis. The summarized results are presented in Table 1.

**Table 1:** Seropositivity percentage and univariate analysis results across individual level variables.

Variable	Level	*N*	Positive	Percentage	95% CI	OR (95% CI)	*p* value
Type	Heifers	33	1	3.03%	(0.00–8.14%)	Ref.	
	Cows	851	21	2.47%	(1.53–3.42%)	0.81 (0.16–14.7)	0.8
	Missing	17	0				
Pregnant	No	527	16	3.04%	(1.90–4.53%)	Ref.	
	Yes	352	6	1.70%	(0.57–2.86%)	0.55 (0.2–1.36)	0.2
	Missing	22	0				
Cow status	Dry	78	1	1.28%	(0.00–3.45%)	Ref.	
	Milking	773	20	2.59%	(1.68–3.71%)	2.05 (0.42–37.0)	0.5
	Missing	50	1				
Parity	1	306	8	2.61%	(1.31–4.43%)	Ref.	
	2	194	4	2.06%	(0.52–3.82%)	0.78 (0.21–2.53)	0.7
	3	137	4	2.92%	(0.73–5.40%)	1.12 (0.29–0.34)	0.9
	≥4	169	5	2.96%	(1.18–5.52%)	1.14 (0.34–3.46)	0.8
	Missing	95	1				
Stay on farm	Stay	650	19	2.92%	(1.85–4.20%)	Ref.	0.3
	No	91	1	1.10%	(0.00–2.95%)	0.37 (0.02–1.81)	
	Missing	160	2				
Abortion history	No	828	21	2.54%	(1.57–3.51%)	Ref.	
	Yes	73	1	1.37%	(0.00–3.68%)	0.53 (0.03–2.61)	0.5
Repeat breeder history	No	339	9	2.65%	(1.18–4.17%)	Ref	
	Yes	198	3	1.52%	(0.51–3.26%)	0.56 (0.12–1.92)	0.4
	Missing	364	10				

The results showed *T. gondii* seropositive farms were detected in all districts, there were 15 seropositive farms, seroprevalence at the farm-level was 29.41% (95% CI, 18.71–43%). In the univariate analysis, three variables were associated with *T. gondii* seropositivity, including the number of companion cats (OR = 1.12, *p* = 0.2), presence of stray cats (OR = 3.44, *p* = 0.067), and frequency of cleaning per day (OR = 3.37, *p* = 0.008). The average number of companion cats in the seropositive group was higher than the seronegative group. Additionally, the percentage of seropositivity was higher in the group with presence of stray cats than in the group without stray cats. Interestingly, the frequency of cleaning in one day was strongly significantly associated with *T. gondii* seropositivity. The results showed that seropositive farms had a higher frequency of cleaning per day compared to seronegative farms. The results of univariate analysis of farm-level seropositivity are presented in Table 2. Logistic regression analysis was performed using these three variables. Results showed that the frequency of cleaning per day was a potential risk factor related to *T. gondii* seropositive farms (OR = 2.745, 95% CI, 1.15–8.69, *p* = 0.02). These results are summarized in Table 3.

**Table 2:** Seropositivity percentage and univariate analysis results across farm level variables.

Variable	Level	*N*	Positive	Percentage	95% CI	OR (95% CI)	*p* value
Hospital	KPS (0)	21	6	22.22%	(11.11–39.32%)	Ref.	
	NP (1)	30	9	23.07%	(12.82–37.28%)	1.07 (0.32–3.80)	>0.9
District	Kamphangsean(1)	11	3	27.27%	(9.09–55.00%)	Ref.	
	Thamaka (2)	11	3	27.27%	(9.09–55.00%)	1.00 (0.14–6.92)	>0.9
	Banpong (3)	13	5	38.46%	(15.38–62.23%)	1.67 (0.30–10.5)	0.6
	Tha Muang (4)	6	3	50.00%	(33.33–98.55%)	2.67 (0.33–23.8)	0.4
	Ban Pong (5)	10	1	10.00%	(0.00–26.53%)	0.30 (0.01–2.86)	0.3
Province	Nakhonpathom(1)	11	3	27.27%	(9.09–55.00%)	Ref	
	Kanchanaburi (2)	17	6	35.29%	(17.64–59.71%)	1.45 (0.29–8.58)	0.7
	Ratchaburi (3)	23	6	26.08%	(13.04–45.68%)	0.94 (0.19–5.36)	>0.9
Farming period	Continuous variable	51	15	Mean 16.5	SD=9.2	1 (0.93–1.07)	>0.9
Cooperative	Nakhonpathom (1)	8	2	25.00%	(12.50–60.17%)	Ref.	
	Sirichok (2)	8	3	37.50%	(12.50–70.93%)	1.80 (0.21–18.3)	0.6
	Thamuang (3)	7	3	42.85%	(14.28–76.44%)	2.25 (0.26–24.0)	0.5
	Nong Pho (4)	28	7	25.00%	(10.71–39.73%)	1.00 (0.18–7.89)	>0.9
Farm standard	No (0)	15	4	26.67%	(13.33–52.19%)	Ref.	
	Yes (1)	36	11	30.56%	(16.67–44.96%)	1.21 (0.33–5.12)	0.8
Total cattle	Continuous variable	51	15	Mean 52.3	SD=21.4	1 (0.98–1.01)	0.7
Farm type	Free stall (0)	36	10	27.78%	(16.67–43.95%)	Ref.	
	Tie stall (1)	15	5	33.33%	(13.33–56.33%)	1.30 (0.34–4.70)	0.7
Presence of companion cat	No (0)	24	7	29.17%	(16.67–49.71%)	Ref.	
	Yes (1)	27	8	29.62%	(14.81–46.89%)	1.02 (0.30–3.49)	>0.9
Total companion cat	Continuous variable			Mean 3.9	SD=7.8	1.12 (0.98–1.38)	0.2*
Presence of stray cat	No (0)	24	4	16.67%	(4.17–29.38%)	Ref.	
	Yes (1)	27	11	40.74%	(25.93–61.32%)	3.44 (0.97–14.3)	0.067*
Feed pile	No	31	9	29.03%	(16.13–46.10%)	Ref.	
	Yes	20	6	30.00%	(15.00–51.86%)	1.05 (0.29–3.57)	>0.9
Feed fresh grass	No (0)	17	5	29.41%	(11.76–50.90%)	Ref.	
	Yes (1)	34	10	29.41%	(17.64–46.33%)	1.00 (0.28–3.81)	>0.9
Pasture	No	49	15	30.61%	(18.37–42.96%)	Ref.	
	Yes	2	0	0.00%	(0.00–85.62%)	0	>0.9
Type of drinking water	Tap (0)	10	2	20.00%	(10.00–48.74%)	Ref.	
	Ground (1)	41	13	31.71%	(19.51–46.81%)	1.86 (0.39–13.5)	0.5
Presence of tank	No (0)	38	11	28.95%	(15.79–42.91%)	Ref.	
	Yes (1)	13	4	30.77%	(15.38–59.46%)	1.09 (0.25–4.16)	>0.9
Feeding area	High (0)	35	11	31.43%	(17.14–46.04%)	Ref.	
	Ground (1)	16	4	25.00%	(12.50–49.15%)	0.73 (0.17–2.65)	0.6
Type of floor	Only concrete	13	3	23.08%	(7.69–47.32%)	Ref.	
	Concrete with Soil	3	1	33.33%	(0.00–68.67%)	1.67 (0.06–24.9)	0.7
	Concrete with soil and rubber	4	1	25.00%	(0.00–60.10%)	1.11 (0.005–13.4)	>0.9
	Concrete with rubber	28	10	35.71%	(21.43–55.13%)	1.85 (0.44–9.70)	0.4
	Concrete with sand	3	0	0.0%	(0.00–56.07%)	0	>0.9
Cleaning frequency/Day	Continuous variable			Mean 2.9	SD=1.0	3.37 (1.52–9.71)	0.008*
Introduce new cow (last year)	No (0)	33	10	30.30%	18.18–47.61%	Ref.	
	Yes (1)	18	5	27.78%	11.11–48.47%	0.88 (0.23–3.08)	0.9
Presence of soil or sand in resting area	No	40	12	30.00%	(17.50–44.54%)	Ref.	
	Yes	11	3	27.27%	9.09–55.00%	0.88 (0.17–3.64)	0.9

**Table 3:** Logistic regression analysis of farm level data.

Variable	Coefficient (95% CI)	Standard error	Z values	Wald test *p* value	OR (95% CI)	Log likelihood ratio test *p* value
Intercept	−4.846 (−6.568–−1.787)	1.632	-2.969	0.002	0.022 (0.001–0.167)	…
Total companion cat	0.1182 (−0.062–0.360)	0.118	1.028	0.304	1.125 (0.940–1.434)	0.099
Presence of stray cat No (0=ref.) Yes (1)	0.582 (−0.953–2.213)	0.581	0.738	0.460	1.789 (0.386–9.147)	0.056
Cleaning frequency/Day	1.001 (0.141–2.163)	0.500	2.018	0.043*	2.745 (1.152–8.694)	0.020*

## Discussion

This study revealed a 1.48% prevalence of *T. gondii* infection in dairy cattle from the Western Region of Thailand. This prevalence was lower than that in previous reports from other parts of the country [4, 20–22]. Because the cattle in these previous reports included young animals (aged 0–1 year), this might be due to the effect of maternal immunity on their high seropositive results. Therefore, the lower *T. gondii* prevalence in this study may be influenced by the difference in the type of animal sample.

Previous studies in Thailand used the LAT for *T. gondii* antibody detection [4, 20–22]. Both IgG and IgM antibodies in serum were able to be agglutinated by parasite antigen in the LAT method. The IgM antibodies could be detected via nonspecific antigens from whole *T. gondii* lysates soluble in LAT. The conserved pathogen-associated molecular patterns (PAMPs) across species were close to those of *T. gondii*, which can react with IgM in serum. Therefore, this reaction could be a false-positive result for LAT [39]. In contrast, in the present study only IgG antibodies were detected, via the P30 (SAG1) antigen. Moreover, comparing the performance of the IDvet ELISA kit and the IFAT showed 82.48% sensitivity and 97.8% specificity [30]. The sensitivity and specificity of LAT when compared with those of the IFAT were 100% and 91.3%, respectively [45]. Because ELISA has a lower sensitivity and higher specificity than LAT, the percentage of positive results would be lower for ELISA. Thus, the low prevalence of *T. gondii* infection in this survey may be caused by the difference in the methods used for antibody detection.

The seroprevalence of *T. gondii* differs in various parts of the world, and this study found lower values than in other countries in South East Asia. The seroprevalence values were reported to be 2.59–6.3%, 7–9%, and 6.6% from Malaysia, Indonesia, and Myanmar, respectively [2, 6, 8, 31, 34, 37]. Reviews of *T. gondii* infections in South Asian cattle, including those from India, Pakistan, and Bangladesh, revealed seropositivity of 42% (CI, 31–49%), 25% (CI, 16–33%), and 12% (CI, 2.5–31%), respectively. The overall percentage of seropositivity from these countries was 27.9% [24]. All these countries surrounding Thailand conducted surveys in beef cattle, which are raised under different management practices compared to dairy cattle. Beef cattle typically roam in free-range pastures, whereas dairy cattle receive more intensive care. For example, a study from India showed that the seroprevalence of free-ranging mithuns (*Bos frontalis*) was 42%, which is higher than captive mithuns, 28% [35, 36]. China is the largest country in the region and has conducted extensive surveys on *T. gondii* in dairy cattle. Some reported seroprevalence rates were similar to those in our study, at 1.93% for the central part of China [12]. However, seroprevalence rates for other parts of China were higher, ranging from 4.87% to 13.71% [44, 46, 52]. This variation in *T. gondii* prevalence was dependent on diverse geographic and local climate conditions and environments.

Furthermore, South America has tropical climates and dairy production similar to Thailand. Previous studies have reported *T. gondii* seroprevalence in dairy cattle ranging from 8.48% to 32% [3, 18]. Also, herd prevalence ranged from 93% to 100%, which is higher than in this study [11, 18]. The weather and environment in Brazil are similar to those in Thailand, but the *T. gondii* seroprevalence is greater than that in Thailand. Because the Brazil dairy industry has a large intensive farming model and is combined with grazing pasture, the possibility of exposing infective oocysts in the environment is increased. Additionally, this area contains numerous wild felids that are in close contact with pasture and drinking water sources [11].

The individual factors of cattle including type, pregnancy status, cow status, and parity exhibited no relation with *T. gondii* infection. There was no difference in seroprevalence between young and old cattle. However, some reports indicated a significant increase in seroprevalence among young cattle; it also increased in older cattle [1, 17, 25, 46]. It is possible that young cattle may show greater susceptible to *T. gondii* infection, and their immune response is stronger than older cattle [16]. Furthermore, the high seroprevalence in older cattle may be attributed to prolonged exposure to *T. gondii* infection [1, 42]. In addition, owing to the results of this cross-sectional study, the association between age and antibody response should be monitored in the long-term in individual cattle.

Our study examined reproductive problems, including abortion history and repeat breeder cattle, but these results did not reveal an association between these two reproductive problems and positivity for *T. gondii* antibodies. Similarly, there was no significant relationship between infection with *T. gondii* in cattle in Brazil and abortion history, even though the prevalence of *T. gondii* was high [28]. Additionally, research in India and China showed that the prevalence of *T. gondii* infection in cattle was not correlated with abortion [19, 44]. Even though infected cattle appear to be resistant to toxoplasmosis, a study in Iran showed that the percentage of *T. gondii*-positive cattle with a history of abortion was significantly greater than that of cattle without a history of abortion [15]. Therefore, the severity of toxoplasmosis in cattle should be investigated to determine the virulence of protozoa in circulation from different areas.

The percentages of seropositive farms in Nakhonpathom, Kanchanaburi, and Ratchaburi Provinces were 20%, 40%, and 40%, respectively. There were positive farms identified from all observed districts, indicating widespread exposure of dairy cattle to *T. gondii*. The services provided by hospitals and cooperatives, as well as standard farm management practices, did not differ significantly in their association with *T. gondii* infection. Because cattle are infected via digestion of the sporulated oocyst of *T. gondii*, previous studies have shown that companion cats and stray cats are risk factors for *T. gondii* infection in dairy cattle [28, 41]. Our univariate analysis revealed factors for which the *p* value was equal to or less than 0.2, including the number of companion cats and the presence of stray cats. However, the multivariate analysis of these two factors revealed no significant associations with *T. gondii*-positive farms. Additionally, the likelihood ratio test of the presence of the stray cat factor was closely related. A report from Midwestern Brazil showed that *T. gondii* infection in beef cattle was not associated with the presence of cats on farms, but wild felids were the most important key to transmitting protozoa [28]. Briefly, the dairy cattle in Thailand were raised in a smallholder production system with a basic level of biosecurity, and companion and stray cats were allowed free contact with the house, feed, and drinking water of the cattle. This free-roaming behavior of cats increases the possibility of *T. gondii* infection via ingestion of sporulated oocysts or tissue cysts from intermediate hosts [50]. In addition, the prevalence of *T. gondii* infection in stray cats was greater than that in companion cats, suggesting that the risk posed by stray cats could lead to contamination of feed and water for dairy cattle or transmission to companion cats through oocysts [28, 29]. Thus, stray cats might be one of the factors influencing *T. gondii* circulation in this area.

Interestingly, the frequency of cleaning per day was associated with the number of *T. gondii* seropositive farms (OR = 2.74; 95% CI, 1.15–8.69; *p* = 0.043). Farmers usually clean the floor before milking twice a day. The cleaning protocol involved using high-pressure water and sweeping to remove feces. This study revealed a high frequency of water used for cleaning associated with *T. gondii* seropositive farms. Due to the prolonged environmental survivability of oocysts, coupled with cleaning procedures, their dissemination across the farm is facilitated. This might be the cause of the increased chance of cattle being infected with *T. gondii*. In addition, it is generally known that felines can shed more than 100 million oocysts per cat [38]. A previous study demonstrated that severity of toxoplasmosis in cattle depends on the inoculation dose of oocyst [14]. The infective dose of *T. gondii* that induced antibody development in cattle included 1.0 × 10^4^–10^6^ oocysts [7, 9, 13]. Thus, it might be that a small number of cats were able to produce enough oocyst to contaminate the environment, sufficiently to maintain disease circulation [19]. The authors consider, even though cats were not associated with *T. gondii* seropositivity, that the frequency of cleaning might increase the possibility of oocyst exposure in dairy cattle and increase *T. gondii* seropositive.

## Conclusions

*Toxoplasma gondii* infection occurred with low seroprevalence which indicated that *T. gondii* continued to circulate in the dairy cow population in the western part of Thailand. This information provided further understanding of farm management systems and *T. gondii* infections on farms. The significant risk factor presented here was the high frequency of cleaning per day that supported the spread and maintenance of infection by *T. gondii.* Although cats were the definitive host of this parasite, they were not identified as a significant risk factor for infection. Moreover, it was recommended to conduct long-term observations of seroprevalence to visualize the dynamic of antibodies in cattle. Additionally, to enhance understanding of the epidemiology of *T. gondii*, similar studies should be carried out in other regions of Thailand.
